# The impact of perceived value on brand image and loyalty: a study of healthy food brands in emerging markets

**DOI:** 10.3389/fnut.2024.1482009

**Published:** 2024-10-03

**Authors:** Elizabeth Emperatriz García-Salirrosas, Manuel Escobar-Farfán, Jorge Alberto Esponda-Perez, Dany Yudet Millones-Liza, Miluska Villar-Guevara, Karla Liliana Haro-Zea, Rodrigo Gallardo-Canales

**Affiliations:** ^1^Grupo de Investigación e Innovación para el Emprendimiento y Sostenibilidad, Universidad Nacional Tecnológica de Lima Sur, Lima, Peru; ^2^Department of Administration, Faculty of Administration and Economics, University of Santiago of Chile, Santiago, Chile; ^3^Faculty of Nutrition and Food Sciences, Universidad de Ciencias y Artes de Chiapas, Tuxtla Gutiérrez, Mexico; ^4^Universidad Tecnológica del Perú, Lima, Peru; ^5^EP de Administración, Facultad de Ciencias Empresariales, Universidad Peruana Unión, Juliaca, Peru; ^6^Facultad de Ciencias de la Ingeniería, Administrativas y Sociales, Universidad Autónoma de Baja California, Tecate, Mexico; ^7^Departamento de Tecnologías de Gestión, Facultad Tecnológica, Universidad de Santiago de Chile, Santiago de Chile, Chile

**Keywords:** perceived value, brand image, loyalty, healthy foods, food industry

## Abstract

**Introduction:**

Food brands that promote a healthy lifestyle are gaining more followers. Healthy food consumers are a conscious and demanding segment that values the quality and benefits they receive from a product and the ethical, environmental, and social impact of their purchasing decisions. The objective of the research is to evaluate the influence of perceived value components on health food brand image and brand loyalty in an emerging market.

**Methods:**

A cross-sectional and explanatory study was conducted considering 612 consumers of a healthy brand in Peru. The participants included women (65.2%) and men (34.8%), with ages between 18 and 56 (M = 22.56; SD = 5.95). Data were collected using a self-report form and statistically analyzed using PLS-SEM.

**Results:**

The study hypotheses confirmed the impact of perceived emotional value, perceived social value, perceived financial value, and perceived quality on brand image and loyalty. However, the proposed model observes that perceived social value has no impact on brand loyalty.

**Conclusion:**

Implementing strategies that help build stronger, healthy brands is part of effective management for business leaders. In this context, the findings indicate that brands should effectively communicate their attributes and offer them that meet and exceed consumer expectations to achieve consumer loyalty. This is a mechanism to consolidate a strong and positive image that facilitates customer loyalty based on perceived value. The results obtained can help marketers and decision-makers in the healthy food industry to design more effective brand strategies, which could increase demand for their healthy products.

## 1 Introduction

Food brands and companies struggle to have the attention of their consumers in a market that is challenged to be increasingly sustainable (1–4). Today, consumers are more aware of a more balanced and healthy diet, which has been considered an essential factor in the inclination towards healthy brands over conventional ones. Brands that offer healthy options can appeal to this growing group of health-conscious consumers. For a food to be considered healthy, it must provide minimal amounts of vegetables, fruits, and whole grains, with strict limits on sodium, added sugar, and saturated fat (4). In this context, consumers of healthy brands have expressed their interest in consuming them and even paying 20% more because they translate it to protect their family’s health and optimize their well-being (5–8).

After the COVID-19 pandemic, the population leaned toward consuming healthy foods that contain solid nutrients capable of preventing diseases. Thus, interest in the consumption of healthy foods was growing due to consumer inclinations, so it has represented a key factor of business growth in the Peruvian market, allowing many brands to awaken new business ideas around healthy foods (9, 10). When issuing an opinion or judgment, these consumers connect the brand they are most familiar with to their feelings (5, 11). Therefore, one of the primary purposes of a marketing campaign is to establish loyalty in its consumers (12–14). Brand loyalty involves the development of a unique and emotional bond with a company through its name, logo, symbols, characters, and slogans throughout the life of consumers (12, 15–17).

The current study recognizes that perceived value has been associated with a substantial nexus between brand loyalty (18–23) and brand image (24–27). The importance of its exploration lies in several factors, one of them being that the market for healthy products is constantly developing (2, 28), with an increasing number of companies competing for the attention of health-conscious consumers. In a competitive market, perceived value becomes a key differentiator. Brands that project superior value can build a solid and unique brand image to stand out in the market. When consumers perceive that a product offers high value compared to its alternatives, consumers are likelier to remain loyal to that brand. Loyalty and preference are critical in healthy products because brand loyalty ensures repeat sales and encourages positive word of mouth and consumer brand advocacy (18, 29–31).

In this context, research on healthy foods has been associated with confidence in purchasing decisions (32), exposure to nature (33), intuitive nutritional labeling (34), health messages (35), willingness to pay (36), emotions (37), culture and health awareness (3), positive emotions (38), price consciousness (39), and mainly with the perceived value (9, 10), loyalty (9, 15, 31, 40) and brand image (41). The importance and interest in these topics have led to previous studies (24, 27, 42–47). However, the impact that the components of perceived value may generate in the context of healthy brands has yet to be explored. Therefore, studying perceived value, brand image, and brand loyalty in an environment of healthy product consumers is a multidisciplinary topic that can provide a more comprehensive approach.

Consequently, after reviewing the background above, a growing interest is revealed in continuing to discern these topics among business leaders, the food industry, and specialists in marketing, consumer psychology, consumer economics, and business strategy. The bibliometric indicators reveal the 10 countries that most disclose scientific results: The United States, China, Indonesia, Taiwan, India, Malaysia, the United Kingdom, South Korea, Australia, and Spain. The same ones have mainly applied their studies to various areas, sectors, and populations, such as business, management and accounting, social sciences, computer sciences, economics, econometrics, and finance. On the other hand, when discerning scientific dissemination by country, research has yet to be found that has evaluated the behavior of these topics in the context of healthy brands in the Peruvian market. In this sense, the objective of the research was to evaluate whether the components of perceived value impact brand image and loyalty.

## 2 Theoretical background

### 2.1 Variable research

#### 2.1.1 Healthy food brand image

Healthy food brand image has gained significant importance in the current context of the food market, where the growing concern for health and well-being has led consumers to seek healthier options in their diet (41). This trend has accelerated in recent years, driven by factors such as the increase in diet-related chronic diseases, increased awareness about nutrition, and the environmental impact of food (48). Keller (49) defines brand image as “the perceptions about a brand reflected by the brand associations retained in the consumer’s memory.” In the context of healthy foods, these associations are strongly linked to nutritional attributes, health benefits, and values related to well-being (50, 51). Healthy food brands must balance the perception of quality and health benefits with affordability to build a positive and sustainable brand image (52).

#### 2.1.2 Healthy food brand loyalty

Brand loyalty refers to a consumer’s deep commitment to consistently repurchase and support a preferred brand of healthy foods (53, 54). Loyalty is characterized by positive brand attitudes and repetitive purchasing behaviours driven by the perception of health and well-being benefits (49, 55, 56). In the health food market, brand loyalty is influenced by factors such as perceived quality, brand trust, perceived authenticity, and alignment with personal values related to health and sustainability (57). Furthermore, brand loyalty is strengthened when consumers perceive that the brand consistently offers functional and experiential benefits, such as taste and satisfaction (58). Importantly, loyalty to healthy food brands may be more resistant to price fluctuations and competition, as consumers value health benefits over purely economic considerations (50, 52). However, this loyalty can also be more demanding since healthy food consumers are more informed and critical of health claims and brand authenticity (28, 59).

#### 2.1.3 Perceived quality

Perceived quality refers to the consumer’s overall evaluation of the excellence or superiority of a product or service (60). Parasuraman et al. (61) state that perceived quality is a consumer judgment about an entity’s overall excellence or superiority. This concept is subjective and can vary significantly between different consumers based on their previous experiences, expectations, and individual contexts. In the context of health food brands, perceived quality refers to the consumer’s overall evaluation of food products’ nutritional excellence, freshness, and purity (62–64). According to Konuk (52), the perceived quality of organic and healthy foods is closely related to the perception of food safety and health benefits. From an economic perspective, perceived quality can justify higher prices, as consumers are willing to pay a premium price for foods they perceive to be of high nutritional quality and free of artificial ingredients (57).

#### 2.1.4 Perceived social value

Perceived social value refers to the utility derived from the product’s ability to enhance the consumer’s social self-concept (65, 66). This concept relates to how consumers perceive that a product or service can improve their social status or image within a group (67–69). Perceived social value in the context of healthy food brands refers to how consuming these products can improve an individual’s social image. Hansen et al. (70) argue that consuming healthy and organic foods has become a form of expression of identity and social status. This implies that consumers may be willing to pay more for products that improve their social image as health and environmentally conscious people (68, 71).

#### 2.1.5 Perceived emotional value

Perceived emotional value is defined as the utility derived from the feelings or affective states that a product generates. Therefore, purchasing decisions are not purely rational but also influenced by emotional factors (65, 66). In the context of healthy foods, the perceived emotional value is related to the positive feelings associated with consuming these products. This can include well-being, satisfaction from caring for one’s health and the planet, and a sense of belonging to a group of conscious consumers. These factors are crucial in shaping food purchase intention (72–74). Organic, since healthy food brands can generate customer loyalty. Kushwah et al. (75) argue that emotional value is closely related to self-actualization and the feeling of doing the right thing. This suggests that brands can leverage these feelings in their communication strategies.

#### 2.1.6 Perceived financial value

Perceived financial value refers to the consumer’s perception of the relationship between the price paid and the value received (10, 60). In economic terms, this concept is closely related to utility and consumer theory. Perceived financial value in the context of healthy foods refers to the consumer’s evaluation of whether the price paid for these products is justified by the benefits received. This includes the direct monetary cost and the potential long-term savings on health expenses. Apaolaza et al. (62) affirmed that consumers who perceive high financial value in organic and healthy foods have higher purchase intentions despite higher prices. This suggests that, in economic terms, health food brands can compete effectively in the market if they can effectively communicate the long-term value of their products (10).

### 2.2 Conceptual model and research hypothesis

According to Keller (49), a positive brand image contributes to the formation of favorable attitudes toward the brand, which fosters loyalty. Furthermore, Batra et al. (55) argue that brand image can evoke positive emotional responses, which is essential for developing brand loyalty. In this context, brand image is a critical factor for loyalty (76, 77). Thus, a strong and positive brand image in healthy foods increases loyalty directly and consumers’ willingness to pay more for products from that brand, thus reinforcing the link between image and loyalty (78). Additionally, Watson et al. (79) proposed that a green brand image mediates the relationship between environmental concern and brand loyalty. Based on the mentioned above, the following hypothesis is proposed:

**H1:** Health food brand image directly and positively influences healthy food brand loyalty.

Environmental values influence attitudes and behaviors (79). Cuesta-Valiño et al. (80) demonstrated the importance of green satisfaction and brand image as antecedents of the green brand value of organic agri-food products. In the same way, Sheth et al. (65) argued that emotional value is one of the five values that influence consumption choices, and in the case of healthy foods, it may be related to feelings of pride in taking care of one’s health and that of one’s family. For example, in the restaurant context, customers’ positive emotional experiences with satisfactory food and service contribute significantly to the formation of loyalty toward these establishments. The study reveals that when diners experience positive emotions such as joy, satisfaction, or enthusiasm during their visit to a restaurant, they are more likely to develop a favorable and lasting attitude towards it.

Singh et al. (81) indicated that emotional value and brand satisfaction are positively related, and both act as significant predictors of brand loyalty for premium and non-premium footwear brands. The brand is the vehicle that leads to establishing and strengthening that emotional, even spiritual, bond that companies must have with their customers (82). Functional value was shown to be more important than emotional value, and information usefulness had relatively greater value compared to other quality dimensions used to determine customer loyalty to the brand (83). Based on the previous context, the following hypotheses are proposed:

**H2:** Perceived emotional value directly and positively influences healthy food brand image.

**H3:** Perceived emotional value directly and positively influences healthy food brand loyalty.

The stronger the brand is, that is, the more recognition and loyalty it has on the part of the customer, or the longer the customer has lived better and more intense experiences with the brand, the brand will have greater brand equity, and consequently will provide greater profitability to the brand (82). In the same way, perceived value is a judgment or evaluation made by the customer comparing the benefits or utility obtained from a product, service, or relationship and the perceived sacrifices or costs (84) Perceived value is the evaluation variable by the consumer. The variable on which companies can work, since later, together with other factors, will result in loyalty behavior on the part of the consumer (85). In this scenario, the perceived value is subordinated to the evaluation judgments of the results obtained, involving pre-purchase information, the quality of services, and brand loyalty (86). Thus, the following hypotheses are proposed:

**H4:** Perceived financial value directly and positively influences healthy food brand image.

**H5:** Perceived financial value directly and positively influences healthy food brand loyalty.

In the context of healthy foods, perceived quality is a critical point that can change a consumer’s general perception of a product. In this way, research supports that a positive perceived quality can build a trustworthy brand image in the minds of consumers (87, 88). The perception of quality is a subjective quality based on belief, knowledge, and experience that could modify the perception of the brand image. That is, when a consumer perceives positive attributes of a brand, he responds by creating a positive image that leads to favorable attitudes such as loyalty (89, 90).On the other hand, previous studies have suggested that perceived quality is a factor that influences brand loyalty positively and directly (91, 92). To explain this association, (147) state that every time a customer experiences a high level of specific expectations and standards for a product, perceived quality assumes a catalytic role in resulting in brand loyalty (93, 94). In this framework, when consumers perceive the excellent quality of a product, their commitment is strengthened, and the tendency to be loyal to the brand increases since it has been proven that quality is crucial in consumers’ purchasing decisions (95).

Service quality is also understood as a precursor to brand loyalty (96) Customer loyalty has become a strategic imperative for most companies. Previous studies conducted in different industries have empirically shown that the overall perception of service quality is one of the essential factors in establishing customer loyalty (96) Customer loyalty has emerged as an essential element of strategy for many firms because strengthening customer loyalty is expected to increase sales results, service quality, and greater profits (97). The importance of measuring loyalty is that the general perception of service quality has been established as one of the factors promoting customer loyalty toward the brand (97) Based on what was mentioned above, the following hypotheses are proposed:

**H6:** Perceived quality directly and positively influences healthy food brand image.

**H7:** Perceived quality directly and positively influences healthy food brand loyalty.

Perceived social value refers to the utility that consumers derive from a product’s ability to enhance their social self-image within a group. In the context of healthy food brands, consuming these products is often associated with a positive social identity since these foods are perceived as responsible and conscious options for personal well-being and the environment. This association can significantly improve brand image, as consumers value brands that allow them to project a positive image in their social circles. Furthermore, previous studies have shown that consumers are willing to pay a higher price for products that improve their social status, reinforcing the relationship between perceived social value and brand image (70). Brand loyalty in the context of healthy foods is influenced by functional factors such as quality or price and social factors. When consumers perceive that a healthy food brand helps them maintain or improve their social status, they are more likely to develop lasting brand loyalty (65). This is particularly relevant in markets where the social image associated with the consumption of healthy products is highly valued (98). Brand loyalty is reinforced when consumers feel that supporting a brand is aligned with its social values, increasing their willingness to make a repeat purchase (98). In this context, the following hypothesis is proposed:

**H8:** Perceived social value directly and positively influences healthy food brand image.

**H9:** Perceived social value directly and positively influences healthy food brand loyalty.

Previous research has shown that brand image is a component that plays an important role in perceived value and loyalty, which is why it deserves to be evaluated exhaustively (99, 100). When accentuating a bond of loyalty between the brand and the consumer, the perceived value assumes a fundamental role that can be strengthened with the intervention of the brand image successful marketing strategy (101, 102). A critical debate on the effect of brand image on increasing consumer loyalty indicates that consumers no longer rely solely on brand image to make repetitive purchases. However, it also evaluates the perceived value. When a consumer believes in a product, has high expectations, and maintains a positive brand image, purchasing behavior becomes repetitive, which translates into loyalty (103, 104).

Likewise, some studies have attributed a special connection between a good brand image and consumer loyalty (101, 105, 106). Other studies have used perceived value (economic, social, emotional value, and quality (9, 107) to drive consumer loyalty (108, 109). In both cases, research has focused on understanding consumer loyalty to deepen and better understand the link between the study variables. Other research reveals that this general evaluation of the consumer (perceived value) precedes loyalty, becoming even more consolidated when the brand image is in the consumer’s mind (110). To explain this, brand image can affect consumer preferences and expectations regarding specific product attributes, driving purchases and repurchases, which translates into loyalty (111).

Likewise, perceived quality is a critical determinant of brand image, especially in the health food market, where consumers associate quality with attributes such as freshness, purity, and nutritional benefits (50, 52). A positive brand image mediated by high perceived quality can lead to greater brand loyalty, as consumers trust that the brand will consistently meet their quality expectations. This mediation process strengthens loyalty through brand trust and continued consumer satisfaction (52). Also, extending the perceived social value as a variable affects brand loyalty through brand image. Consumers who perceive that a brand improves their social status tend to have a more favorable image of the brand, strengthening their loyalty (70). In this sense, brand image bridges social value and loyalty, ensuring consumers continue to prefer the brand that allows them to project a desired social identity. Based on what was mentioned above, the following hypothesis is proposed:

**H10:** Healthy food brand image plays a mediating role in the influence of perceived emotional value on healthy food brand loyalty.

**H11:** Healthy food brand image plays a mediating role in the influence of perceived financial value on healthy food brand loyalty.

**H12:** Healthy food brand image plays a mediating role in the influence of perceived quality on healthy food brand loyalty.

**H13:** Healthy food brand image plays a mediating role in the influence of perceived social value on healthy food brand loyalty.

**Figure 1:**
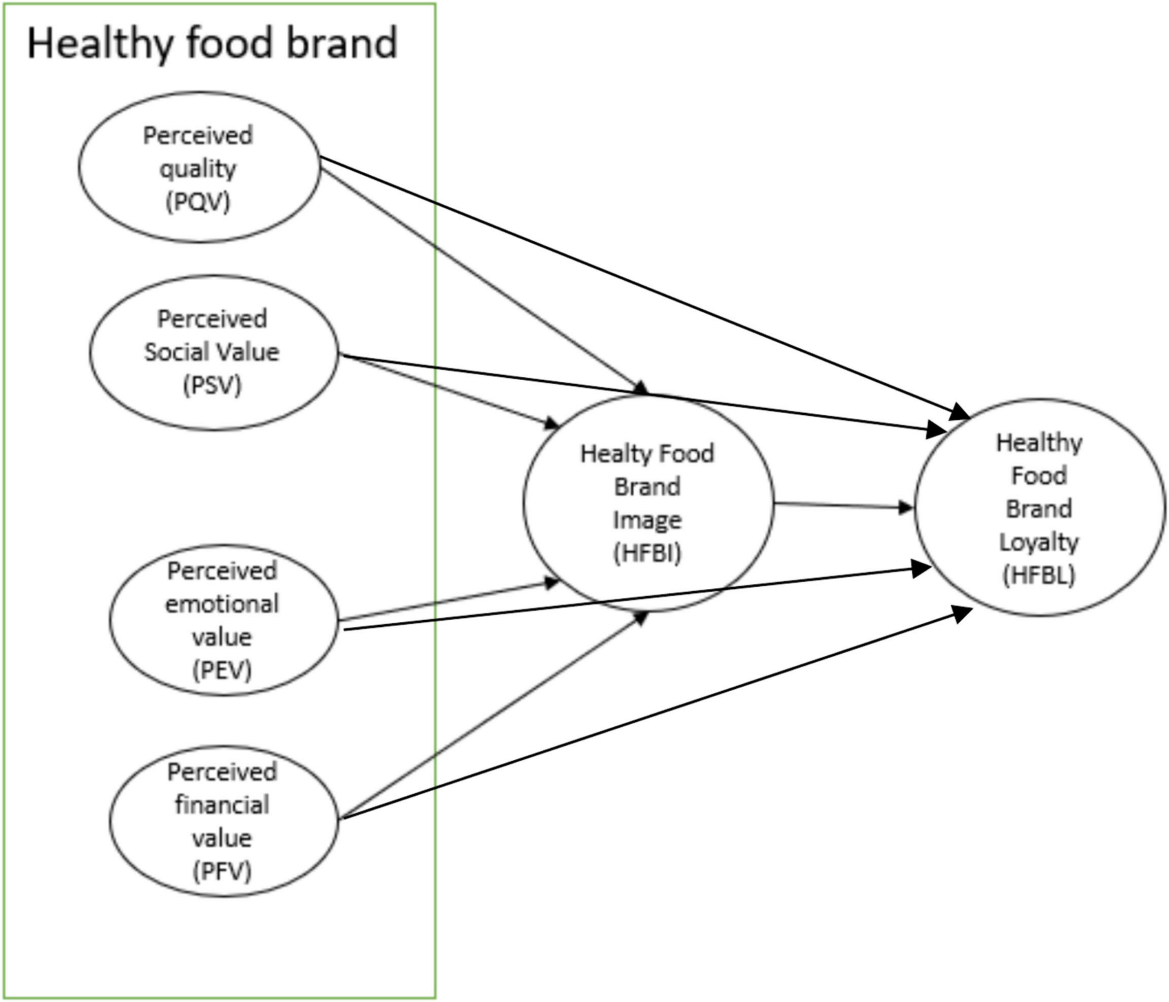
Proposed model.

Considering the hypotheses mentioned above, the conceptual model resulting from the study can be visualized, as represented in Figure 1.

## 3 Materials and methods

### 3.1 Study design and participants

The present investigation has been considered within a cross-sectional and explanatory study (112). The study population comprised Peruvian residents who consumed products from a healthy brand (Marca Unión). The brand that was considered for this study has an essential representativeness in the Peruvian market and currently has a presence in Ecuador, Chile, and the United States; it is valued as a healthy brand that looks after the well-being of its consumers and that manufactures its products based on whole or whole grains. Within the inclusion criteria, participants had to be of legal age (minimum 18 years) and consumers of a healthy brand, excluding all participants who did not meet these requirements.

Non-probabilistic sampling was applied to establish the sample size (113) and the electronic tool Soper was used (114). This online tool considers the number of observed and latent variables in the Structural Equation Model (SEM), along with the anticipated effect size (*λ* = 0.20), the desired level of statistical significance (*α* = 0.05), and the power required statistic (1 − *β* = 0.80). Based on these standards, the need to include a minimum of 296 consumers in the sample was determined. However, a total of 612 people participated, including women (65.2%) and men (34.8%), with ages ranging between 18 and 56 years (M = 22.56; SD = 5.95). Most of the participants were female consumers (65.2%), with an age range between 18 and 22 years (66.3%), single (93.3%), and who declared that they had a higher university education (95.8%), as shown in Table 1.

**Table 1:** Profile sociodemographic of the participants (*n* = 612).

Feature	Category	Frequency	%
Age range	18–22 years	406	66.3
	23–56 years	206	33.7
Sex	Male	213	34.8
	Female	399	65.2
Marital status	Single	571	93.3
	Married	35	5.7
	Cohabitant	2	0.3
	Divorced	4	0.7
Academic training	Regular Basic Education (EBR)	19	3.1
	Technical Superior	7	1.1
	University Higher	586	95.8

### 3.2 Measurement scales

An online questionnaire was designed and divided into three parts to collect the data. In the first section, filling instructions were given. In the second section, the sociodemographic information of the participants was requested, and in the last section, the measurement scales were presented. This research used three reliable measurement scales, which evaluated each item using a 5-point Likert-type scale, rated from 1 (strongly disagree) to 5 (strongly agree). To evaluate the perceived value, a short scale was used (115), which assessed four components: perceived quality value (PQV), perceived social value (PSV), perceived emotional value (PEV), and perceived financial value (PFV). Three items were used for each construct. A short 3-item scale adapted from previous studies was used to measure brand image (89, 116, 117), and brand loyalty was measured in 2 items adapted from previously published studies (89, 117, 118). Each of the items of the questionnaire can be found at the end of the document in Appendix A.

### 3.3 Ethical considerations

The research was approved by the Ethics Committee of the Graduate School of a private university (2023-CE-EPG-00043). Subsequently, between July and December 2023, participants were invited to complete an online questionnaire shared by WhatsApp and Telegram through Google Forms. Before data collection, the study made sure to follow the confidentiality rules and the principles of the Declaration of Helsinki (119, 120), informing the participants about the study’s objective, and obtaining informed consent from each person involved through the following statement: “I agree to participate in this study.”

### 3.4 Statistical analysis

The partial least squares structural equation model (PLS-SEM) was used to perform the statistical analysis of the data. Two stages were carried out to evaluate the PLS-SEM: evaluating the psychometric properties of the measurement scale, such as reliability, convergent, and discriminant validity, and testing hypotheses through the system of structural equations. The choice of PLS-SEM is due to its usefulness when analyzing several dependent, independent and latent variables, in addition, it has the key advantage of simultaneously analyzing the structural model, providing a complete view of the interaction of the variables (121).

## 4 Results

### 4.1 Convergent validity

According to Hair et al. (122), to evaluate the measurement model, an estimate of the reliability of the constructs (composite reliability and Cronbach’s alpha) and validity (discriminant and convergent validity) was proposed. Cronbach’s alpha values are between 0.892 and 0.953, and the threshold value of 0.7 falls below these values (122). Likewise, the composite reliability (CR) shows values between 0.892 and 0.954, which were above the suggested value of 0.7 (123). Based on these findings, all constructs were considered error-free, and construct reliability was established (Table 2).

**Table 2:** Convergent validity results.

Construct	Items	Factor loading	Alpha	CR	AVE
Healthy Food Brand Image (BI)	BI1	0.953	0.946	0.946	0.902
	BI2	0.943			
	BI3	0.953			
Healthy Food Brand Loyalty (BL)	BL1	0.949	0.892	0.892	0.902
	BL2	0.951			
Perceived Emotional Value (PEV)	PEV1	0.952	0.945	0.947	0.900
	PEV2	0.957			
	PEV3	0.937			
Perceived Financial Value (PFV)	PFV1	0.927	0.926	0.927	0.871
	PFV2	0.940			
	PFV3	0.932			
Perceived Quality (PQV)	PQV1	0.958	0.953	0.953	0.914
	PQV2	0.950			
	PQV3	0.961			
Perceived Social Value (PSV)	PSV1	0.958	0.950	0.954	0.909
	PSV2	0.957			
	PSV3	0.945			

The convergent validity results ensured acceptable values [Factor loading, Cronbach’s Alpha, and composite reliability (CR) ≥ 0.70 and Average Variance Extracted (AVE) > 0.5].

Average variance extracted (AVE) and factor loading should be tested for convergent validity (122). According to Table 1, all factor loadings had values above the suggested value of 0.7. Furthermore, Table 2 shows that the AVE returns have values between 0.871 and 0.914 and are above the threshold value of 0.5. With these results, convergent validity for all constructs is adequately satisfied.

### 4.2 Discriminant validity

Two criteria were considered to determine discriminant validity: (1) The Fornell-Larker criterion and (2) the Heterotrait-Monotrait (HTMT) relationship (122). Table 3 confirms the requirements by the Fornell-Larker condition since all AVEs and their square roots are more significant than their correlations with other constructs (124).

**Table 3:** Fornell-Larcker scale.

	BI	BL	PEV	PFV	PQV	PSV
BI	**0.950**					
BL	0.683	**0.950**				
PEV	0.640	0.693	**0.949**			
PFV	0.677	0.714	0.730	**0.933**		
PQV	0.756	0.735	0.692	0.705	**0.956**	
PSV	0.351	0.440	0.605	0.564	0.405	**0.953**

The diagonal values in bold represent the square of the average variance extracted (AVE).

Table 4 provides the results of the HTMT relationship, which shows that the threshold value of 0.85 is greater than the value of each construct (125). These findings determine discriminant validity. These results confirm the validity and reliability of the measurement model, allowing the evaluation of the structural model to continue.

**Table 4:** Heterotrait-monotrait (HTMT) relationship.

	BI	BL	PEV	PFV	PQV	PSV
BI						
BL	0.743					
PEV	0.676	0.755				
PFV	0.722	0.785	0.779			
PQV	0.796	0.797	0.728	0.749		
PSV	0.369	0.478	0.638	0.600	0.424	

### 4.3 Structural model analysis

The PLS-SEM method was used to compare the suggested hypotheses. For model fitting, predictive relevance values were employed. The predictive relevance of the model is represented by cross-validated redundancy values (R2). For a model to be accurate, its R2 values need to exceed “0” (125, 126). Using the blindfolding method, R2 values were calculated. All endogenous construct values were more significant than 0, indicating the correctness of the model. Table 5 displays the endogenous latent variables together with corresponding R2.

**Table 5:** R2 of the endogenous latent variables.

Construct	R2
Healthy Food Brand Image (BI)	0.623
Healthy Food Brand Loyalty (BL)	0.645

**Figure 2:**
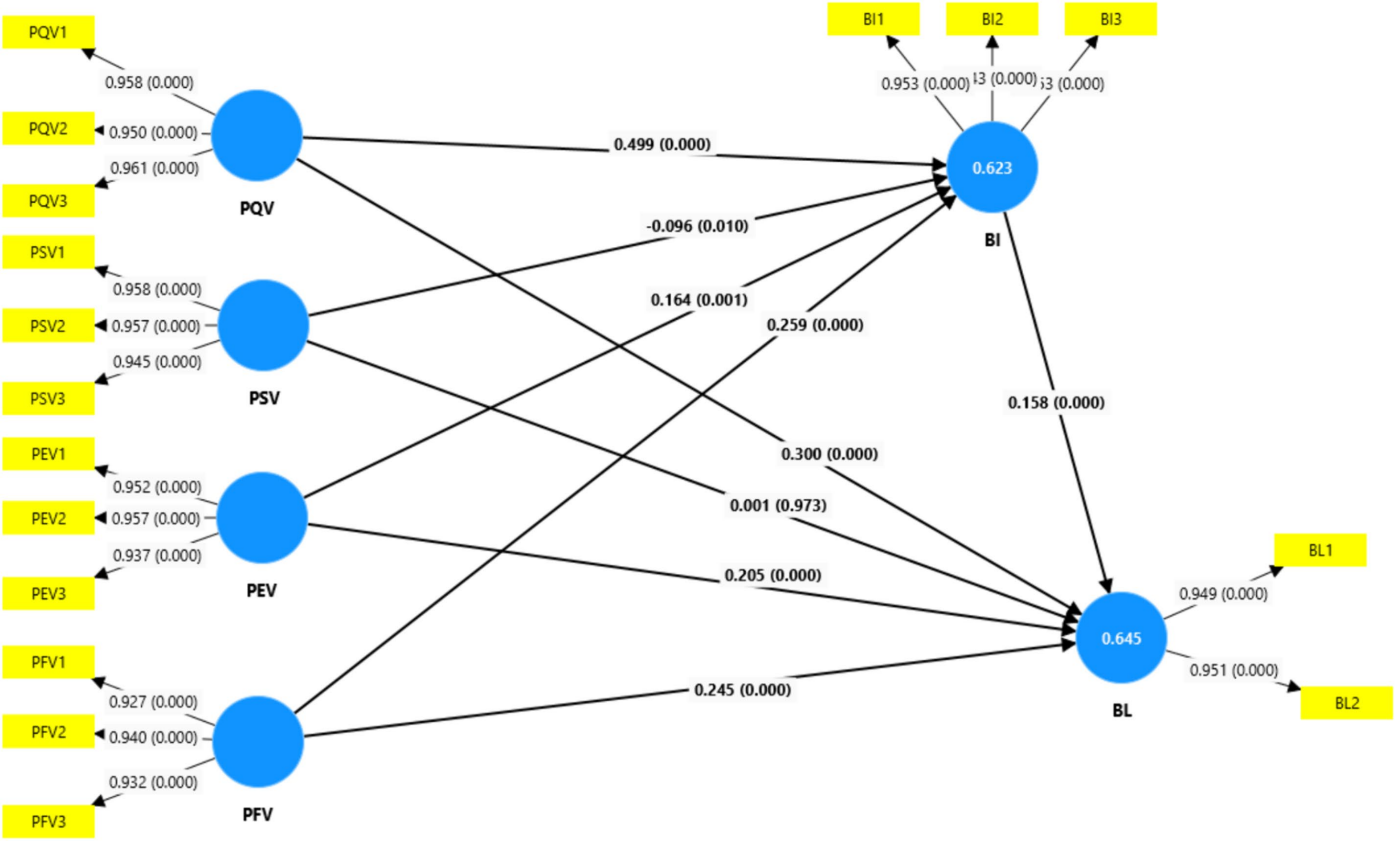
Structural model.

As seen in Figure 2 and Table 6, the path coefficient values, *p*-value, and *t*-statistics were utilized to accept and reject the hypotheses. The strength of the association between the variables can be examined using the coefficient values. Path. Strong relationships are indicated by path coefficient values near +1, and vice versa (127). The acceptance and rejection of the put forward hypotheses are shown by the *p* values and the *t* statistics. In this study, the conceptual model contains 13 hypotheses. The results of the tested hypotheses have been summarized in Table 6. H1 is accepted, which proposed that healthy food brand image (BI) has a positive impact on healthy food brand loyalty (BL) (*β* = 0.158, *p* = 0.000, *t* = 3.496); H2 was accepted, which proposed that perceived emotional value (PEV) has a positive impact on healthy food brand image (BI) (*β* = 0.164, *p* = 0.001, *t* = 3.215); H3 is accepted, which proposed that perceived emotional value (PEV) has a positive impact on healthy food brand loyalty (BL) (*β* = 0.205, *p* = 0.000, *t* = 4.008).

**Table 6:** Structural model results.

h	Hypothesis	Original sample (O)	Sample mean (M)	Standard deviation (STDEV)	T statistics (|O/STDEV|)	*p*-values	Decision
H1	BI—BL	0.158	0.160	0.045	3,496	0.000	Supported
H2	PEV—BI	0.164	0.164	0.051	3,215	0.001	Supported
H3	PEV—BL	0.205	0.206	0.051	4,008	0.000	Supported
H4	PFV—BI	0.259	0.261	0.047	5,507	0.000	Supported
H5	PFV—BL	0.245	0.243	0.050	4,935	0.000	Supported
H6	PQV—BI	0.499	0.497	0.049	10,293	0.000	Supported
H7	PQV—BL	0.300	0.299	0.049	6,085	0.000	Supported
H8	PSV—BI	−0.096	−0.096	0.038	2,564	0.010	Supported
H9	PSV—BL	0.001	0.000	0.037	0.034	0.973	Not supported
H10	PEV—BI—BL	0.026	0.026	0.010	2,480	0.013	Supported
H11	PFV—BI—BL	0.041	0.042	0.015	2,761	0.006	Supported
H12	PQV—BI—BL	0.079	0.080	0.024	3,257	0.001	Supported
H13	PSV—BI—BL	−0.015	−0.015	0.007	2,155	0.031	Supported

H4 is accepted, which proposed that perceived financial value (PFV) has a positive impact on healthy food brand image (BI) (*β* = 0.259, *p* = 0.000, *t* = 5.507); H5 is accepted, which proposed that perceived financial value (PFV) has a positive impact on healthy food (BL) brand loyalty (*β* = 0.245, *p* = 0.000, *t* = 4.935); H6 is accepted, which proposed that perceived quality (PQV) has a positive impact on healthy food brand image (BI) (*β* = 0.499, *p* = 0.000, *t* = 10.293); H7 is accepted, which proposed that perceived quality (PQV) has a positive impact on healthy food brand loyalty (BL) (β = 0.300, *p* = 0.000, *t* = 6.085); H8 is accepted, which proposed that perceived social value (PSV) has an impact positive in the image of the healthy food brand (BI) (*β* = −0.096, *p* = 0.010, *t* = 2.564); H9 is not accepted, which proposed that perceived social value (PSV) has a positive impact on healthy food (BL) brand loyalty (*β* = 0.001, *p* = 0.973, *t* = 0.034); H10 is accepted, which proposed that healthy food brand image (BI) has a mediating role in the influence of perceived emotional value (PEV) on healthy food brand loyalty (BL) (β = 0.026, *p* = 0.013, *t* = 2.480); H11 is accepted, which proposed that healthy food brand image (BI) has a mediating role in the influence of perceived financial value (PFV) on healthy food brand loyalty (BL) (β = 0.041, *p* = 0.006, *t* = 2.761); H12 is accepted, which proposed that healthy food brand image (BI) has a mediating role in the influence of perceived quality (PQV) on healthy food brand loyalty (BL) (β = 0.079, *p* = 0.001, *t* = 3.257) and H13 is accepted, which proposed that healthy food brand image (BI) has a mediating role in the influence of perceived social value (PSV) on healthy food brand loyalty (BL) (β = −0.015, *p* = 0.031, *t* = 2.155).

## 5 Discussions

Peruvian macroeconomic stability has led to its classification as a country with an emerging economy. This means that the economy is going through a phase of accelerated growth and development (128), this fact is attributed, in part, to the emergence of innovative businesses within the healthy food sector and that after the arrival of the pandemic, trends in the consumption of healthy foods have been increasing (10, 28, 129). The increase mentioned above has prompted the commercial sector to make decisions to satisfy demand, a demand that promotes public health, an aspect increasingly valued by consumers (98, 130). This entire scenario has become an opportunity for companies in this sector to focus their strategies on promoting consumer loyalty. Consequently, it improves your brand image and reinforces the value consumers perceive.

In this context, this study focused on analyzing the factors of perceived value and the intervention of brand image to achieve consumer loyalty. In this context, it has been demonstrated in the first instance that perceived value influences the brand image of healthy foods. To support this result, the background establishes that a better perception of a brand is attributed to the one that generates benefits and sustainability, such is the case of healthy foods that have a special value for their impact on the lifestyle and well-being of individuals (109). Other research that supports the results found is focused on antecedents that support that healthy food brands maintain an optimal perceived value, this is due to the ideological barriers that significantly contribute to the consumption of natural foods, traditional products to which a higher perceived value is attributed compared to processed products; Under this scenario, it is demonstrated that, despite the time that has passed, healthy foods continue to occupy an essential place in the consumer’s life, thus generating a positive image, which is crucial to encourage repurchase intentions (131, 132).

Furthermore, the results of this research are further supported by the statement that establishes the importance of the contribution of the general evaluation that a consumer makes towards a brand’s products in economic, social, emotional, and quality terms towards the brand image, which gives rise to its lasting interaction between the consumer and the brand, thus causing the feeling of trust and commitment that allows a brand to maintain a positive reputation in the market (133–135). In other words, it is stated that brand image tends to improve when the perceived value is greater (136, 137); therefore, disseminating positive and authentic consumer experiences through social platforms and word-of-mouth marketing becomes a favorable strategy to mobilize brand image through perceived value (30).

Likewise, the findings reflect that perceived value influences brand loyalty to healthy foods. One of the supports that support this result is that every brand must offer authentic value to consumers to generate benefits that generate positive behavioral results, taking into account that every perception of a product or brand is built based on quality, price, and emotional attachment and when this is positive and goes beyond the consumer’s expectations, loyalty behavior is developed. Lasting, translating this into repetitive purchases and recommendations of the product and/or brand (138, 139). In addition, another idea that supports the results establishes that consumer demand for healthy foods has allowed consumers to maintain a positive perception, which becomes an opportunity to strengthen the bond of the brand with the consumer, giving openness to a solid and lasting loyalty (108). Likewise, it supports that a reasonable adjustment of consumer perceptions enhances constant purchase intention until consolidating long-term loyalty (13, 140).

Another of the results obtained establishes that in the population of the study, perceived social value does not influence brand loyalty to healthy foods. A study that supports this finding makes it clear that affective factors and purchase intention are more determining factors than rational thinking when promoting consumer loyalty, this suggests that emotions, quality, and price have a high contribution in consumer loyalty, in contrast to social value that does not seem to have an essential link in loyalty (13, 140). When it comes to consuming healthy foods, the most important thing is the tangible benefit it generates (prevention of diseases), and the social value or social status falls behind the priorities (10, 141).

Based on the results found in this study, it is stated that brand image assumes a mediating role in the influence of perceived value toward brand loyalty. A study that supports these results supports that brand image is a factor that can alter consumer behavior, which means that every company must dedicate its efforts to improve the brand image to reduce dilemmas and stimulate consumer loyalty and that the brand image, beyond transmitting information and experience, represents an important factor in the connection between perceived value and consumer loyalty (142, 143). Likewise, previous studies that support the results indicate that companies should analyze other factors, such as the customer’s perceived value, beyond focusing on satisfaction as a predictor of loyalty, to obtain an explanation. More updated on how to get more loyal customers, also taking into account another factor such as brand image, the one in charge of mediating this association (144, 145), so it is conclusive that brand image strengthens the effect of perceived value on consumer loyalty (17, 146). Consequently, to achieve consumer loyalty, brands should effectively communicate their attributes and offer them by covering and exceeding consumer expectations. This is a mechanism to consolidate a strong and positive image that serves as a facilitator in customer loyalty based on perceived value.

### 5.1 Theoretical and practical implications

Analyzing the impact of perceived value and brand loyalty in the health food industry in emerging markets has significant theoretical and practical implications. Theoretically, this study enriches the existing literature on consumer behavior and healthy food marketing by highlighting the influence that brand image has on purchase intention for nutritious products in developing countries. Incorporating perceived emotional value, perceived quality, and brand image in this context opens new horizons in food marketing research, focusing on aspects such as brand personality. This provides a novel perspective on how consumers perceive and emotionally connect with healthy food brands.

In practical terms, this research’s results provide valuable information and have concrete applications. The insights gained can help marketers and decision-makers in the healthy food industry design more effective brand strategies, which could increase demand for their healthy products. By better understanding how consumers emotionally value and perceive quality and brand image, marketers can adapt their strategy to strengthen brand loyalty in emerging markets.

### 5.2 Management implications

The implications for management in the context of healthy food purchase intention and the crucial role of brand image are of great relevance. Managers must recognize the importance of cultivating a favorable brand image when formulating marketing strategies to increase consumer preference for healthy foods. In this context, managers must invest in developing a strong and positive brand image. This can be achieved through various strategies, such as effective advertising campaigns, public relations initiatives, sponsorship of events that promote health, and the participation of brand ambassadors who highlight the benefits of the products. Through these actions, consumers can perceive healthy foods as more beneficial options for their well-being and lifestyle.

Likewise, the permanent monitoring that managers must carry out of how consumers perceive the brand image becomes more relevant to adapting their marketing strategies in response to timely market changes. This continuous monitoring will allow strategies to be adjusted to maintain the relevance and attractiveness of the brand among consumers. In addition, managers must consider specific market characteristics in developing countries, such as health consciousness, local taste preferences, and consumer purchasing power. Adapting marketing strategies to these characteristics can facilitate the creation of a greater preference for healthy foods and avoid consumer rejection.

Finally, the Ministries of Health and Education can play a fundamental role in promoting healthy lifestyles. Implementing practical courses and programs promoting healthy eating and lifestyle can inspire and motivate new generations to have a broader perspective and prefer healthy food brands. Collaboration between the public and private sectors on these initiatives can amplify the impact of marketing efforts and contribute to a cultural shift toward a preference for more nutritious foods. In summary, managing healthy food purchase intention and promoting a positive brand image requires a strategic and adaptive approach. By investing in building a solid brand image, monitoring consumer perceptions, and adapting strategies to market characteristics, managers can increase consumer preference for healthy foods. Furthermore, collaboration with public institutions can enhance these efforts, contributing to a significant change in consumer habits and the general well-being of the population.

### 5.3 Limitations of the study

The study used a cross-sectional design, which limits the ability to establish definitive causal relationships between perceived value, brand image, and loyalty. Future studies could consider a longitudinal design to observe how these relationships evolve. The sample focused on specific emerging markets. Although relevant to the study context, the results may not be fully generalizable to other markets or regions with different socioeconomic and cultural characteristics. It is suggested that future studies replicate the study in other geographies to evaluate the generalizability of the findings.

Data collection was based on self-administered surveys, which could have introduced response biases, such as social desirability or recall bias. Future research could benefit from triangulation of methods, including in-depth interviews or observational data, to mitigate these biases. Although the study addressed several dimensions of perceived value, other relevant variables were not considered, such as the influence of competition, product characteristics, or the economic context. This could have influenced the results, and future research could have included these variables to obtain a more complete picture.

Finally, despite the high participation of the study population, no differences have been analyzed with respect to their perspectives on the topic under investigation, since no homogeneous characteristic was achieved to measure gaps between different groups, so it is proposed as future research to deepen the comparative analysis between the various subpopulations in order to identify possible discrepancies and factors that influence their perceptions.

### 5.4 Future research

As mentioned above, it is recommended that future studies use a longitudinal design to investigate how brand value perception and brand image evolve and how this affects consumer loyalty. While this study focused on healthy food brands, it would be valuable to explore whether the same patterns are observed in other sectors, such as technology or financial services, to evaluate the applicability of the results in different industries. Future studies could explore how contextual variables, such as economic conditions, marketing campaigns, or market competition, affect the relationship between perceived value, brand image, and loyalty.

Expanding the research to include multicultural analysis could shed light on how cultural differences influence value perception, brand image, and loyalty. This would be especially relevant in an increasingly globalized world. The role of possible mediating and moderating variables that were not addressed in this study could be further explored. For example, investigate whether brand trust or emotional commitment mediate between perceived value and loyalty.

## 6 Conclusion

The present study has provided a comprehensive view of how different components of perceived value influence brand image and consumer loyalty towards health food brands in emerging markets. The findings underline the importance of emotional, financial, and quality value as determining factors in building a solid brand image and generating customer loyalty.

In particular, perceived quality and financial value were confirmed to be the biggest drivers of positive brand image and brand loyalty, indicating that consumers value product quality and value for money in their decision-making. On the other hand, although emotional value also shows a significant influence, its effect is smaller than the other two components, suggesting that, although emotions play a relevant role, economic rationality and product quality are more determinants in customer loyalty.

A noteworthy aspect of this study is that, unlike the other dimensions of perceived value, social value did not show a direct impact on brand loyalty, which could indicate that, in the context of health food brands in Peru, social perceptions are not a critical factor in the decision to continue buying a particular brand. However, its influence on brand image suggests that it could be relevant to the brand’s overall perception, although not necessarily in direct loyalty.

The mediating role of brand image was also confirmed, especially in the relationship between perceived emotional, financial, and quality value and loyalty. This reinforces that a strong brand image is essential for translating perceived values into loyalty behaviors, functioning as a bridge between consumer perception and action.

The results of this research can guide brand managers in the health food industry to develop strategies that improve the quality and perceived value of their products and strengthen brand image to ensure consumer loyalty. It also suggests the need to focus marketing strategies on highlighting product quality and value for money without neglecting the emotional component to consolidate a lasting relationship with consumers. Finally, although social value does not appear to be a direct factor in loyalty, it should not be ignored entirely, as it contributes to the brand’s overall perception.

## Data Availability

The raw data supporting the conclusions of this article will be made available by the authors, without undue reservation.

## Appendix A

**Table A1:** Questionnaire used for the study.

Healthy Food Brand Image (BI)		The Union brand.
	BI1	Has a good reputation
	BI2	Addresses my health concerns
	BI3	Is reliable
Healthy Food Brand Loyalty (BL)		Personally I …
	BL1	Prefer the Union brand over other brands of healthful products
	BL2	Recommend the Union brand to my friends and family
Perceived Emotional Value (PEV)		Consuming Union brand products…
	PEV1	Gives me satisfaction
	PEV2	Makes me feel good
	PEV3	It gives me peace of mind
Perceived Financial Value (PFV)		Buying Union brand products…
	PFV1	Offer good value for money
	PFV2	They are worth the price
	PFV3	Are reasonably priced
Perceived Quality (PQV)		Union brand products…
	PQV1	Are always of good quality
	PQV2	Have a good presentation
	PQV3	Have an adequate shelf life
Perceived Financial Value (PFV)		Consuming Union brand products…
	PEV1	Gives me satisfaction
	PEV2	Makes me feel good
	PEV3	Gives me peace of mind
Perceived Social Value (PSV)		Consuming Union brand products…
	PSV1	Helps me feel accepted by others
	PSV2	Improves the way I am perceived
	PSV3	Gives me social approval
